# PD-1 inhibitor sintilimab treated patients with metastatic triple-negative breast cancer

**DOI:** 10.3389/fcell.2024.1430310

**Published:** 2024-10-14

**Authors:** Yan Jia, Jie Zhang, Yehui Shi, Guolei Dong, Xiaojing Guo, Zhongsheng Tong

**Affiliations:** ^1^ Department of Breast Oncology, Tianjin Medical University Cancer Institute and Hospital, Tianjin, China; ^2^ Tianjin’s Clinical Research Center for Cancer, Tianjin, China; ^3^ Key Laboratory of Breast Cancer Prevention and Therapy, National Clinical Research Center for Cancer, Tianjin Medical University, Ministry of Education, Tianjin, China; ^4^ Department of Breast Pathology and Lab, Tianjin Medical University Cancer Institute and Hospital, Tianjin, China

**Keywords:** immune checkpoint inhibitor, sintilimab, triple-negative breast cancer, immunotherapy, tumor microenvironment

## Abstract

**Purpose:**

Triple-negative breast cancer (TNBC) is a highly challenging subtype due to a unique tumor microenvironment. Several evidence (IMpassion130 trial and KEYNOTE-355 trial) supported the therapeutic effect of the immune checkpoint inhibitor in TNBC. However, the efficacy and safety of the PD-1 inhibitor sintilimab in breast cancer (BC) has not been well-investigated. So the real-world data on sintilimab-treated patients with metastatic BC were collected and analyzed in this study.

**Methods:**

The patients were eligible according to the requirements included: ages between 18 years and 75 years; recurrent or metastatic TNBC; measurable disease based on RECIST v1.1; no limitation on the prior systemic treatments; and ECOG performance status of 0–1. Patients received sintilimab 200 mg intravenously every 3 weeks until unacceptable toxicity or disease progression.

**Results:**

From 1 June 2019 to 1 October 2022, 40 female patients (median age, 55.5 years) with metastatic TNBC (mTNBC) were enrolled into the study. The median prior lines of systemic therapy for mTNBC was three (range, 1–8), with 60% of cases receiving at least three lines of therapy for metastatic disease. The visceral or brain metastasis was detected in 40.4% or 9.6% of patients, respectively. The median duration of response was 2.8 months (range, 0.7–21.0), and the median number of sintilimab doses administered was 4 (range, 1–30). The ORR and DCR were 22.5% and 72.5%, separately. The median PFS was 3.5 months (range, 1.4–21.0), with a 6-month PFS rate of 15.0% (6/40). The median OS was 52.5 months (range, 9.0–247.0) as of data cut-off. Common adverse effects were acceptable, and fatigue, skin rash, and pruritus were the frequent toxicity observed. Two cases of grade 3 curable adverse events were considered to be treatment-related. PD-L1-positive tumor was found in 40% cases (4/10) of mTNBC. Although statistical difference was not reached, the trend was obvious. Patients with PD-L1-positive tumor gained better treatment response, while the TMB-high carrier received more benefits of PFS and OS.

**Conclusion:**

In our study, preliminary evidence provided the anticancer activity and acceptable adverse effects of sintilimab administered every 3 weeks to pretreated patients with mTNBC. Sintilimab showed its efficacy and safety of immunotherapy for patients with advanced TNBC.

## Introduction

Female breast cancer has surpassed lung cancer as the most diagnosed cancer, with an estimated 2.3 million new cases (11.7%) worldwide in 2020. It is also the leading cause of female cancer death (Sung et al., 2021). Breast cancer is a significant public health issue in China with incidence and mortality rates increasing rapidly since the 1980s (Li et al., 2016). In addition, 416,371 Chinese women were diagnosed with breast cancer in 2020 (Tao et al., 2023).

Breast cancer is a highly heterogeneous disease. Triple-negative breast cancer (TNBC) represents up to 20% of all breast cancers. It is histologically defined by a lack of estrogen receptor and progesterone receptor expression and the absence of human epidermal growth factor receptor 2 (HER2) overexpression and/or amplification (Foulkes et al., 2010). TNBC is also a highly challenging subtype of breast cancer due to a unique tumor microenvironment (TME). TME associates with its aggressive nature, metastatic potential, drug resistance, and poor prognosis in TNBC (Deepak et al., 2020). The mortality rate of TNBC is 40% within the first 5 years after diagnosis (Yin et al., 2020). Compared to other types of breast cancer, TNBC has limited treatment options. It is not sensitive to endocrine therapy or anti-HER2 targeting therapy. The standardized treatment regimens of TNBC are still lacking, so development of new treatment strategies has become an urgent clinical need.

TME is highly complex; the suppression of immune system and evasion of immune surveillance are intrinsically linked to it (Hanahan and Coussens, 2012; Hanahan and Weinberg, 2011). The programmed cell death receptor-1 (PD-1) is upregulated on activated T cells and binds two known ligands: programmed death ligand-1 (PD-L1) and programmed death ligand-2 (PD-L2). Through interactions with PD-L1 on the surface of tumor cells and immune cells, PD-1 signaling counters T-cell activation during the effector phase of the immune response (Pardoll, 2012). In recent years, immunotherapy is revolutionizing the management of multiple solid tumors including breast cancer. Some solid tumors that harbor stromal-infiltrating immune cells (TILs) and express PD-L1 are more likely to respond to PD-1/PD-L1 blockade, which suggests that patients with TNBC may obtain the clinical benefit from immunotherapy. Several previous data revealed the clinical activity of PD-1/PD-L1 antagonists, e.g., pembrolizumab and atezolizumab in patients with metastatic breast cancer (Emens, 2018). Sintilimab (Tyvyt^®^) is a fully human IgG4 monoclonal antibody that binds to PD-1, thereby blocking the interaction of PD-1 with its ligands and consequently helping restore the endogenous antitumor T-cell response (Hoy, 2019; Zhang et al., 2020). It has been approved by the National Medical Products Administration of China to treat relapsed or refractory classical Hodgkin lymphoma in patients who have undergone two or more lines of systemic chemotherapy and combine with pemetrexed and platinum-based chemotherapy for the first-line treatment of non-squamous non-small cell lung cancer (nsqNSCLC) (Hoy, 2019; Zhang et al., 2020). However, the antitumor effect and safety of sintilimab in advanced triple-negative breast cancer have not been well-investigated.

In this study, we aim to collect real-world data and explore the efficacy and safety of sintilimab, providing a novel therapeutic strategy for patients with mTNBC, especially for the ones pretreated for several lines.

## Materials and methods

### Design and treatment

The study was designed to evaluate the antitumor activity, tolerability, and safety of sintilimab in patients with advanced TNBC. The eligible requirements were as follows: ages between 18 years and 75 years; estrogen receptor-negative, progesterone receptor-negative, and HER2-negative in the metastatic setting, recurrent, or metastatic breast cancer, respectively; measurable disease per Response Evaluation Criteria in Solid Tumors (RECIST) version 1.1 (Eisenhauer et al., 2009); an Eastern Cooperative Oncology Group (ECOG) performance status of 0–1; any number of prior systemic treatments.

Patients received sintilimab 200 mg intravenously every 3 weeks until unacceptable toxicity or disease progression. No limitation on the combination of chemotherapy. If the efficacy was satisfied and safety was acceptable, six cycles of combinational treatment were administered to patients. For the subsequent cycles, only sintilimab was given to avoid accumulated adverse effects from chemotherapy. In case of clinically partial response or stable disease, patients with first radiologic evidence of disease progression were permitted to continue sintilimab until a second scan performed 3 weeks later confirmed progression. The study was approved by the institutional review boards and ethics committees at the Tianjin Medical University Cancer Institute and Hospital. All patients provided written informed consent.

### Efficacy and safety

The data on the overall response rate (ORR), disease control rate (DCR), progression-free survival (PFS), and overall survival (OS) were all analyzed in this study. Patients evaluable for response were those with measurable disease at baseline, who received at least one dose of sintilimab, and who had at least one postbaseline scan or discontinued therapy before the first scan as a result of progressive disease (PD) or a treatment-related adverse event (AE).

AEs were graded by the Common Terminology Criteria for Adverse Events (CTCAE) version 5.0 during the treatment and for up to 30 days thereafter. Serious AEs were collected for up to 90 days after the last administration of sintilimab. The regular monitoring of laboratory assessments, vital signs, and physical examinations was arranged for the safety of patients. Imaging was performed every 6 weeks, after two doses of sintilimab, and response was evaluated based on RECIST v1.1 assessed by experienced radiological experts.

### Immunohistochemistry

The expression of PD-L1 was assessed in formalin-fixed, paraffin-embedded tumor samples using a standard immunohistochemistry assay with PD-L1 antibody (Cell Signal Technology, MA). Positivity was defined as PD-L1 expression in the stroma or in ≥1% of tumor cells. The stained result was evaluated by experienced pathological experts.

### Statistical analysis

The Kaplan–Meier method was performed for analysis of survival. The t-test and chi-squared test were applied to explore the association between clinical outcomes and characteristics of patients or tumors.

## Results

### The characteristics of patients

From 1 June 2019 to 1 October 2022, 40 female patients were enrolled into the study. All patients were confirmed as TNBC in metastatic setting, regardless of the subtypes of primary breast cancer, and most of them were heavily pretreated for advanced diseases. The baseline characteristics of patients are shown in Table 1. The median age was 55.5 years (range, 28–75 years). The main type of pathology is invasive ductal carcinoma, accounting for 82.5% of patients. Other pathological types included invasive lobular carcinoma (7.5%) and metaplastic carcinoma (10.0%).

**TABLE 1:** Baseline of patient information and clinical characteristics.

Characteristic	Value
Age, years	Median (range)
	55.5 (28–75)
Female	No. (%)
	40 (100.0)
ECOG performance status	No. (%)
0	18 (45.0)
1	22 (55.0)
Pathological type	No. (%)
Invasive ductal carcinoma	33 (82.5)
Invasive lobular carcinoma	3 (7.5)
Metaplastic carcinoma	4 (10.0)
Expression of ER^a^	No. (%)
<1%	29 (72.5)
≥1%	11 (27.5)
Expression of PR^a^	No. (%)
<1%	29 (72.5)
≥1%	11 (27.5)
HER2^a^	No. (%)
HER2-negative (HER2 0)	27 (67.5)
HER2-low (IHC 1+ or IHC 2+/FISH-negative)	7 (17.5)
HER2-positive (IHC 2+/FISH-positive or HER2 3+)	6 (15.0)
Expression of Ki67^a^	No. (%)
≤30%	12 (30.0)
>30%	28 (70.0)
Preliminary TNM stages	No. (%)
Stage I	8 (20.0)
Stage II	8 (20.0)
Stage III	19 (47.5)
Stage IV	5 (12.5)
Grade	No. (%)
Grade I	3 (7.5)
Grade II	18 (45.0)
Grade III	19 (47.5)
Location of metastases	No. (%)
Lymph node	26 (22.9)
Lung	19 (16.7)
Liver	15 (13.1)
Thoracic wall	15 (13.1)
Brain	11 (9.6)
Bone	11 (9.6)
Serosa and serous effusion	10 (8.8)
Breast	3 (2.6)
Pancreas	1 (0.9)
Bladder	1 (0.9)
Skin and soft tissue	1 (0.9)
Eye socket	1 (0.9)
Previous neoadjuvant or adjuvant therapy	No. (%)
	35 (87.5)
No. of prior therapies for metastatic disease	No. (%)
Median (range)	3 (1–8)
≤2	16 (40.0)
3	11 (27.5)
4	5 (12.5)
≥5	8 (20.0)
Previous therapy exposure for metastatic disease	No. (%)
Taxane	35 (18.1)
Platinum	31 (16.1)
Capecitabine	29 (15.0)
Vinorelbine	27 (14.0)
Anti-angiogenesis	23 (11.9)
Gemcitabine	17 (8.8)
Anthracycline	14 (7.3)
Cyclophosphamide	5 (2.6)
Etoposide	5 (2.6)
Eribulin	4 (2.1)
Irinotecan	2 (1.0)
Pemetrexed	1 (0.5)

^a^ primary tumor.

The molecular subtypes of primary breast cancer contained luminal A, luminal B (HER2-positive and HER2-negative), HER2-overexpression, and TNBC. The subtypes of all non-TNBC patients were changed into TNBC when breast cancer relapsed or metastatic, based on the pathological confirmation of metastatic tumors. For the molecular subtypes of primary breast cancer during early stage, 27.5% of patients had high expression of estrogen receptors (ER, ≥1%) or progesterone receptors (PR, ≥1%). The ratios of patients with different HER2 expression were 67.5% for HER2-negative (HER2 0), 17.5% for HER2-low (IHC 1+ or IHC 2+/FISH-negative), and 15.0% for HER2-positive (IHC 2+/FISH-positive or HER2 3+). In addition, 70.0% of patients had high expression of Ki67 (>30%). Five cases of patients (12.5%) were diagnosed with *de novo* stage IV breast cancer. According to the tumor-node-metastasis staging system (TNM staging system), the proportion of patients with breast cancer of stage I, stage II, and stage III was 20.0%, 20.0%, and 47.5%, respectively. Breast cancer of most patients (92.5%) was histologically grade II and grade III. For the locations of cancer metastasis, 9.6% of patients suffered from brain metastasis including cerebrum and cerebellum, and 40.4% of patients had visceral metastases including lung (16.7%), liver (13.1%), serosa and serous effusion (8.8%), pancreas (0.9%),and bladder (0.9%). Non-visceral metastases were found located in lymph nodes, thoracic wall, breast, eye socket, skin, and soft tissue (Table 1).

Thirty-five patients (87.5%) were initially diagnosed with early-stage disease and had received neoadjuvant or adjuvant therapy. In the metastatic setting, all patients had received at least one line of therapy. The median number of prior lines of systemic therapy for metastatic disease was 3, ranging from 1 to 8, with 60% of patients having received at least three lines of therapy for metastatic disease and 20% having received at least five lines before treatment of sintilimab. Considering the previous therapy exposure for metastatic diseases, taxane, platinum, capecitabine, vinorelbine, anti-angiogenesis, gemcitabine, anthracyclines, cyclophosphamide, etoposide, and eribulin were the top 10 most applied drugs.

### Antitumor activity

The efficacy was assessed in all patients who received at least one dose of sintilimab, had measurable disease at baseline per RECIST v1.1, and had either at least one postbaseline scan or discontinued the treatment as a result of progressive disease or a treatment-related AE before the first scan. The data on efficacy, overall response rate (ORR), and disease control rate (DCR) were all summarized in this study.

**FIGURE 1:**
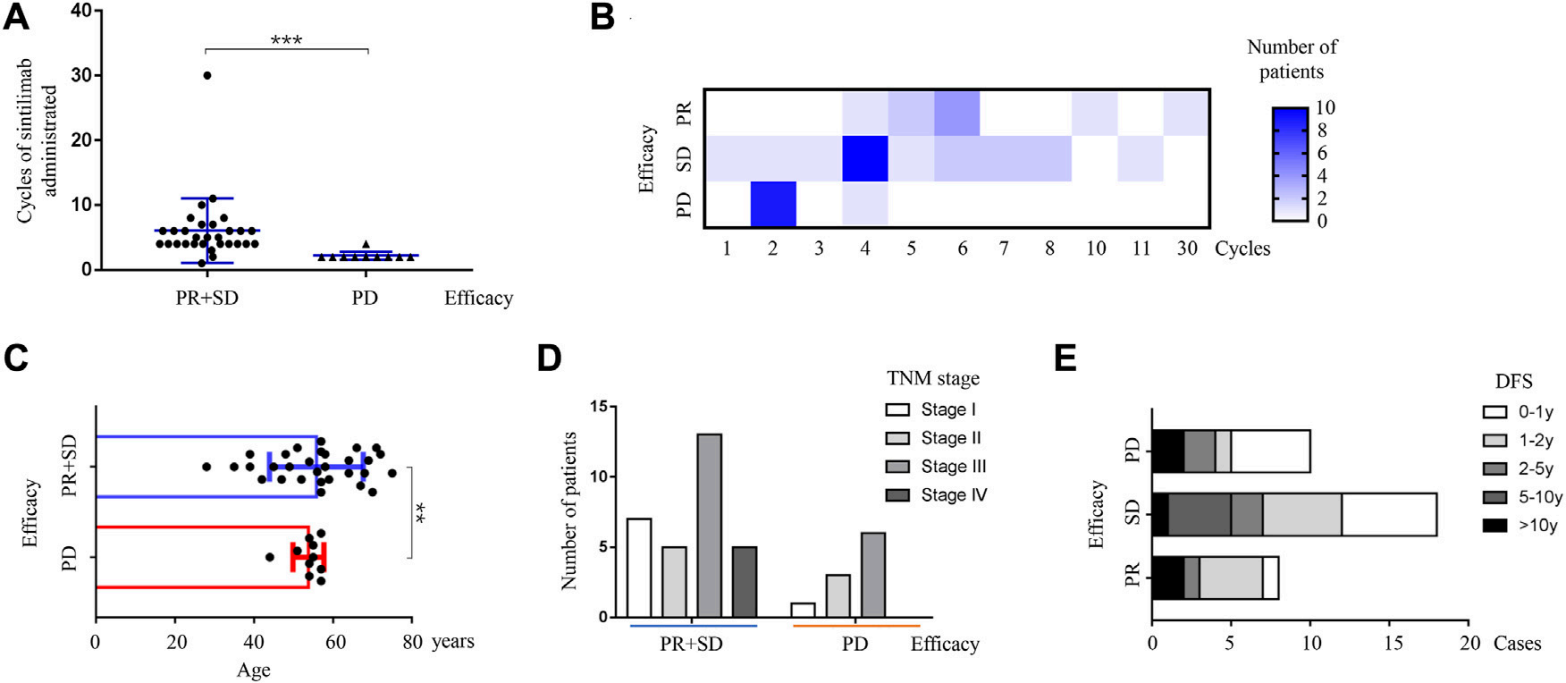
Correlation between the efficacy of sintilimab and characteristics of patients or tumors. **(A)** Efficacy of sintilimab was significantly associated with cycles of administration (*p* < 0.001, t-test). Two groups of favorable and unfavorable treatment responses were shown on the X-axis. The favorable treatment response included partial response and stable disease. The unfavorable treatment response was progressive disease. The blue horizontal bars represented medians for each group. Cycles of sintilimab administered were demonstrated on the Y-axis. PR, partial response; SD, stable disease; PD, progressive disease; no CR in this study; *p < 0.05; **p < 0.01; ***p < 0.001. **(B)** Relationship between the efficacy of sintilimab and cycles of administration was indicated as a heatmap. Heatmap visualization of patients in PR, SD, and PD groups. Each column represented different cycles of sintilimab administered. Each row represented different efficacies of treatment including PR, SD, and PD. The number of patients was illustrated by legends with different colors (from white to blue). The darker the blue, the more patients in the group. The maximal cycle of sintilimab administered was 30 as of data cut-off. PR, partial response; SD, stable disease; PD, progressive disease. **(C)** The age of patients was significantly related to the efficacy of sintilimab. A better treatment response of sintilimab was observed in the elder population compared with the young ones (*p* = 0.0015, t-test). Ages ranging from 0 to 80 years were shown on the X-axis. Two groups of efficacy including favorable and unfavorable treatment responses were shown on the Y-axis. The favorable treatment response included PR and SD. The unfavorable treatment response was PD. The blue and red vertical lines and bars represented mean ages and standard deviation for each group. PR, partial response; SD, stable disease; PD, progressive disease; **p < 0.01. **(D)** The TNM stage of primary breast cancer was not significantly related to the efficacy of sintilimab (*p* > 0.05, chi-squared analysis). Two groups of efficacy including favorable and unfavorable treatment responses were shown on the X-axis. The favorable treatment response included PR and SD. The unfavorable treatment response was PD. The number of patients was demonstrated on the Y-axis. The columns with different colors represented different TNM stages including stage I, stage II, stage III, and stage IV. PR, partial response; SD, stable disease; PD, progressive disease. **(E)** Efficacy of sintilimab was not affected by disease-free survival of patients with breast cancer (*p* > 0.05, chi-squared analysis). The cases of patients were shown on the X-axis. Three groups of efficacy including PR, SD, and PD were shown on the Y-axis. Different DFS was shown with different colors in each row. PR, partial response; SD, stable disease; PD, progressive disease; DFS, disease-free survival; 0–1 y: 0–1 year; 1–2 y: 1–2 years; 2–5 y: 2–5 years; 5–10 y: 5–10 years; >10 y: more than 10 years.

As shown in Table 2, the median duration of response was 2.8 months (range, 0.7–21.0 months), and the median number of sintilimab doses administered was 4, ranging from 1 to 30 doses. The efficacy of sintilimab was obviously related to doses administered (*p* < 0.001, Figures 1A, B). The association between efficacy and cycles of sintilimab given was demonstrated as a heatmap. Patients with treatment response of PD received no more than four cycles of administration. For the best overall response, we observed 9 cases (22.5%) of partial response (PR), 21 cases (52.5%) of stable disease (SD), 10 cases (25.0%) of progressive disease (PD), and none of complete response (CR). The ORR of sintilimab-treated patients with advanced TNBC was 22.5%. The DCR was found to be 72.5% in this study. All patients exhibited long-lasting PR or SD to the treatment of sintilimab, even if several prior lines of systemic therapy were already given for metastatic disease.

**TABLE 2:** Efficacy of sintilimab-treated patients with advanced TNBC.

Response type	Patients evaluable for response, N = 40
Overall response rate, %	22.5%
Disease control rate, %	72.5%
Best overall response, No. (%)	
Complete response (CR)	0
Partial response (PR)	9 (22.5%)
Stable disease (SD)	21 (52.5%)
Progressive disease (PD)	10 (25.0%)
Median cycle of sintilimab treated^a^, range	4 (1–30)
Median duration of response (months)^a^, range	2.8 (0.7–21)

^a^ as of data cut-off.

The definition of favorable results represented therapeutic response of CR (complete response), PR (partial response), and SD (stable disease). Because there was no CR detected in our study, so treatment responses of PR and SD meant to be favorable. Meanwhile, the treatment response of progressive disease (PD) meant to be unfavorable. As illustrated by Figure 1C, the age of patients at the time of administration was significantly related to the efficacy of sintilimab. The mean age of patients with efficacy of PR and SD was 55.8 ± 2.2, while the mean age of patients with efficacy of PD was 53.8 ± 1.2. The elder patients had a significantly better treatment response (*p* = 0.0015). For the TNM staging system, the size of primary tumor, metastasis of the regional lymph node, and distant organ are well-known risk and prognostic factors for patients with breast cancer. Except for five patients diagnosed with *de novo* stage IV breast cancer, primary tumors and axilla or sentinel lymph nodes were removed by surgery during the early stage of breast cancer. Thus, the description of lymph nodes in this study was generated from axilla lymph nodes instead of metastatic sites. It was found that the status of primary tumor and metastasis of the lymph node or distant organ were not significantly associated with the efficacy of sintilimab (all *p* > 0.05, data not shown). Based on the efficacy including favorable (PR + SD) and unfavorable (PD) groups, the treatment effect of sintilimab was not influenced by the TNM stage of primary breast cancer (*p* > 0.05, Figure 1D). In addition, the efficacy of sintilimab was not affected by disease-free survival (DFS) of patients (*p* > 0.05, Figure 1E), which suggested that the status of early-stage breast cancer, following neoadjuvant or adjuvant therapy, was not an important factor to predict the benefit from sintilimab treatment in the advanced stage.

**FIGURE 2:**
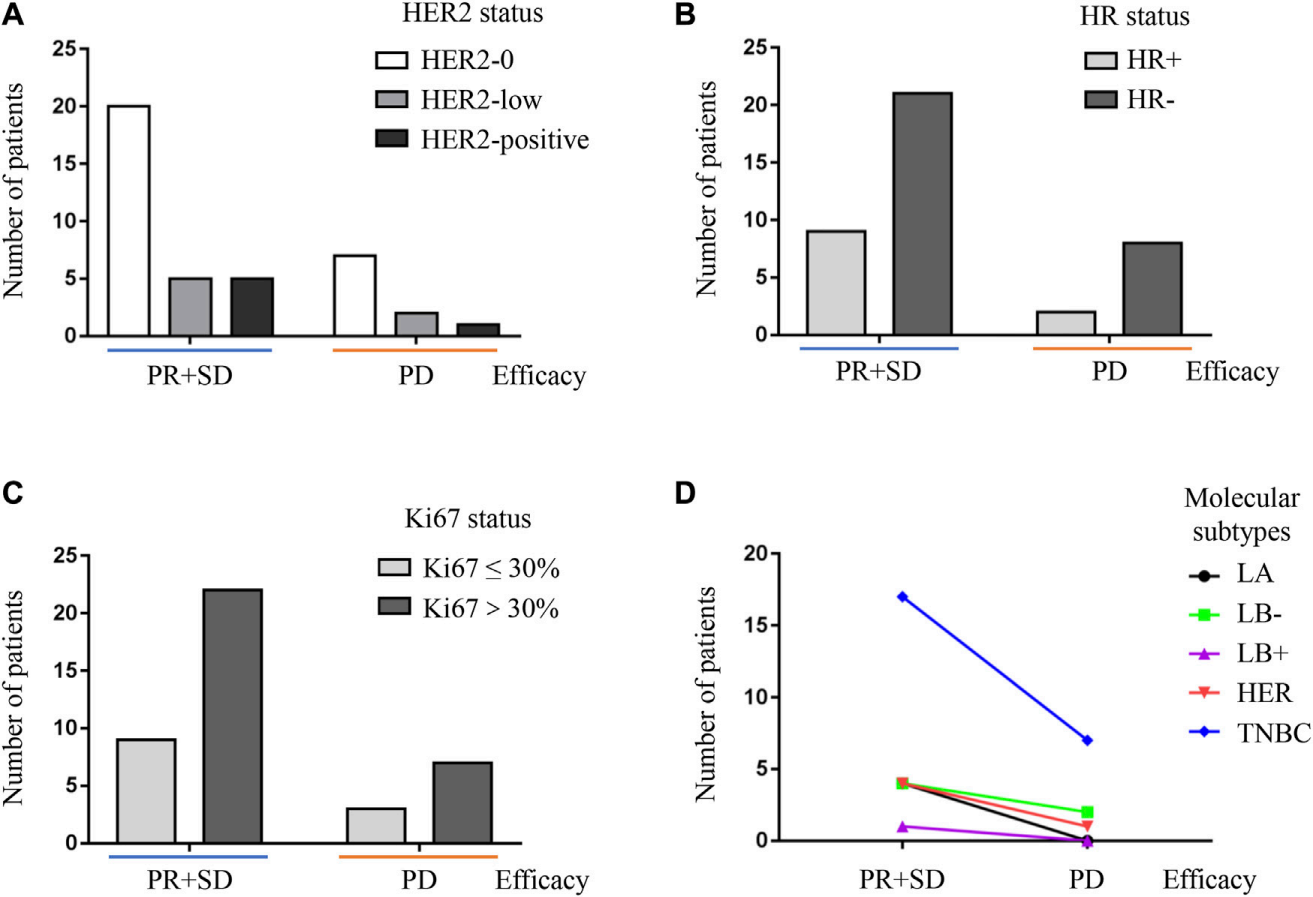
Correlation between the efficacy of sintilimab and molecular markers of primary breast cancer. **(A)** Efficacy of sintilimab was not influenced by different expression of HER2 (*p* > 0.05, chi-squared analysis). Two groups of efficacy including favorable and unfavorable treatment responses were shown on the X-axis. The favorable treatment response included PR and SD. The unfavorable treatment response was PD. The number of patients was demonstrated on the Y-axis. The expression of HER2 contained three different statuses which was HER2 0, HER2 low, and HER2 positive, and was shown as different columns with different colors. PR, partial response; SD, stable disease; PD, progressive disease; HER2-0: HER2-negative breast cancer; HER2-low: IHC 1+ or IHC 2+/FISH-negative breast cancer; HER2-positive: IHC 2+/FISH-positive or HER2 3+ breast cancer. **(B)** The expressed status of hormone receptor had no impact on the therapeutic effect of sintilimab (*p* > 0.05, Chi-square analysis). Two groups of efficacy including favorable and unfavorable treatment responses were shown on the X-axis. The favorable treatment response included PR and SD. The unfavorable treatment response was PD. The number of patients was demonstrated on the Y-axis. The expression of HR contained two different statuses, which was HR-positive (HR+, defined as ER/PR ≥ 1%) and HR-negative (HR-, defined as ER/PR<1%), and was shown as different columns with different colors. PR, partial response; SD, stable disease; PD, progressive disease; HR+, hormone receptor-positive breast cancer; HR-,hormone receptor-negative breast cancer. **(C)** The therapeutic response of sintilimab was not affected by the expressed level of Ki67 with the cut-off value of 30% (*p* >0.05, chi-squared analysis). Two groups of efficacy including favorable and unfavorable treatment responses were shown on the X-axis. The favorable treatment response included PR and SD. The unfavorable treatment response was PD. The number of patients was demonstrated on the Y-axis. The expressed level of Ki67 contained two different statuses which was Ki67-high (Ki67 > 30%) and Ki67-low (Ki67 ≤ 30%) and was shown as different columns with different colors. PR, partial response; SD, stable disease; PD, progressive disease; Ki67 ≤ 30%: Ki67-low breast cancer; Ki67 > 30%: Ki67-high breast cancer. **(D)** The efficacy of sintilimab was not influenced by different subtypes of primary breast cancer (*p* > 0.05, chi-squared analysis). Two groups of efficacy including favorable and unfavorable treatment responses were shown on the X-axis. The favorable treatment response included PR and SD. The unfavorable treatment response was PD. The number of patients was demonstrated on the Y-axis. Molecular subtypes of primary breast cancer contained five different types, which were luminal A breast cancer, luminal B with HER2-negative breast cancer, luminal B with HER2-positive breast cancer, HER2 overexpressed breast cancer, and triple-negative breast cancer and demonstrated as different lines with different colors. PR, partial response; SD, stable disease; PD, progressive disease; LA, luminal A breast cancer; LB-, luminal B with HER2-negative breast cancer; LB+, luminal B with HER2-positive breast cancer; HER, HER2 overexpressed breast cancer; TNBC, triple-negative breast cancer.

All patients received administration of sintilimab based on the pathological confirmation as TNBC of metastatic tumors. Considering the molecular subtypes of primary breast cancer were not the same (shown as Table 1), the correlation between the efficacy of sintilimab and molecular features including HER2 expression, hormone receptor (HR) status, and the Ki67 level was all analyzed in this study. As indicated in Figure 2, it was demonstrated that the treatment response of sintilimab was not influenced by HER2 expression, HR status, and the Ki67 level of primary breast cancer. No significant difference was detected from the statistical analysis of above molecular markers (all *p* > 0.05, Figures 2A–C). Patients with primary TNBC, compared to those with other subtypes of primary tumors, gained an obvious trend of efficacy benefit; however, the statistical difference was not reached (*p* > 0.05, Figure 2D). The conclusion from our small enrolled population was that the treatment response of sintilimab was not depending on different subtypes of primary breast cancer.

### Safety and tolerability

**FIGURE 3:**
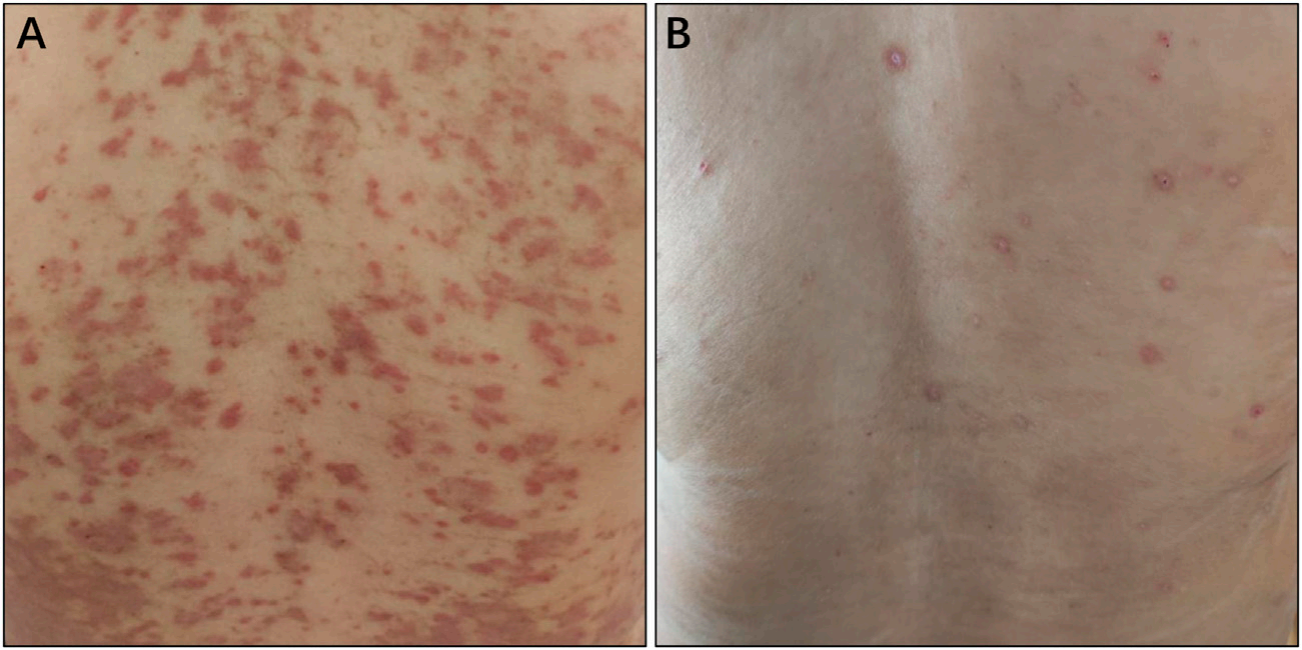
Treatment-related skin rash. **(A)** Skin rash was shown to spread widely over the whole back of the patient. **(B)** In some patients, skin rash was isolated on the local area of the body. The isolated rash was shown on the back of the patient.

The safety was evaluated in all patients who received at least one dose of sintilimab. Overall, 16 cases (40%) of patients experienced at least one treatment-related toxicity. Only one patient (2.5%) experienced grade 3 adverse effects. No grade 4 adverse event was found in this study. As shown in Table 3, the most common treatment-related AEs of any grade contained fatigue (17.5%), skin rash (7.5%), pruritus (5.0%), increased ALT (7.5%), increased AST (7.5%), myalgia (5.0%), arthralgia (5.0%), hyperbilirubinemia (5.0%), immune-mediated hepatitis (2.5%), suppurative cholangitis (2.5%), nausea (2.5%), and headache (2.5%). Based on our clinical observation, the quality of life was affected by the symptoms of skin rash with pruritus. The skin rash of sintilimab commonly occurred in the back of the body, as demonstrated in Figure 3. The skin rash with pruritus was relieved and cured with the help of oral steroids guided by an experienced dermatologist. The grade 3 treatment-related AEs were observed including immune-mediated hepatitis and suppurative cholangitis. Considering the elevated liver function, patients recovered after receiving comprehensive therapy including steroids, choleretics and hepatoprotective medicine. None of the patients died from treatment-related AEs. To sum up, sintilimab had a favorable safety profile during the follow-up.

**TABLE 3:** Treatment-related adverse effects.

Type of adverse effects, N = 40	Grade, No. (%)
Fatigue	Grade 1, 7 (17.5)
Skin rash	Grade 2, 3 (7.5)
Pruritus	Grade 2, 2 (5.0)
ALT increased	Grade 1, 1 (2.5); Grade 2, 2 (5.0)
AST increased	Grade 1, 1 (2.5); Grade 2, 2 (5.0)
Myalgia	Grade 1, 1 (2.5); Grade 2, 1 (2.5)
Arthralgia	Grade 1, 1 (2.5); Grade 2, 1 (2.5)
Hyperbilirubinemia	Grade 1, 1 (2.5); Grade 2, 1 (2.5)
Immune-mediated hepatitis	Grade 3, 1 (2.5)
Suppurative cholangitis	Grade 3, 1 (2.5)
Nausea	Grade 1, 1 (2.5)
Headache	Grade 1, 1 (2.5)

### Survival

**FIGURE 4:**
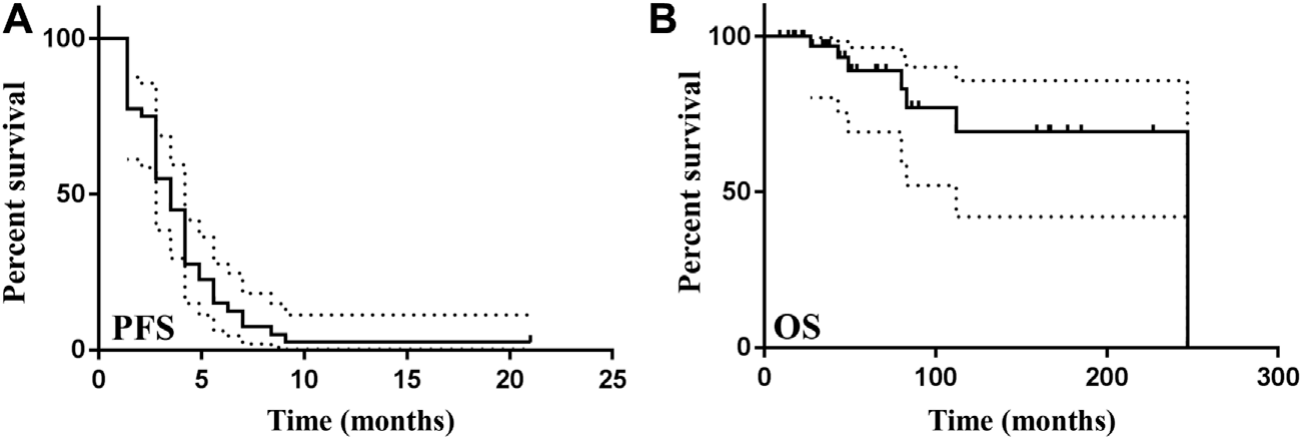
Survival of patients administered with sintilimab. **(A)** The progression-free survival of patients treated with sintilimab was exhibited by the Kaplan–Meier method. The survival time ranging from 0 to 25 months was shown on the X-axis. The percent of survival patients was demonstrated on the Y-axis. The solid line represented PFS of patients. The dashed lines described the error bar of PFS with 95% confidence interval. PFS, progression-free survival. **(B)** The overall survival of patients administered with sintilimab was shown by the Kaplan–Meier method. The survival time ranging from 0 to 300 months was shown on the X-axis. The percent of survival patients was demonstrated on the Y-axis. The solid line represented OS of patients. The dashed lines described the error bar of OS with 95% confidence interval. OS, overall survival.

As the cut-off date of all data was 1 April 2024, the median duration of the follow-up was 11.5 months ranging from 2 to 40 months since the first dose of sintilimab was administered. The progression-free survival (PFS) and overall survival (OS) were both explored in this study. PFS was defined as the time from the first administration of sintilimab to the first occurrence of disease progression or death from any cause (whichever occurs first). OS was defined as the time from the first confirmation of breast cancer to death from any cause. As shown in Figure 4A, 39 PFS events were observed, and median PFS was 3.5 months (range, 1.4–21.0), with a 6-month PFS rate of 15.0% (6/40). One responder remained on the study and had received sintilimab for 21.0 months as of data cut-off. The median OS was 52.5 months (range, 9.0–247.0) as of data cut-off, with 5-year and 10-year OS rates of 47.5% (19/40) and 22.5% (9/40), respectively (Figure 4B).

### Alterations of genes

**FIGURE 5:**
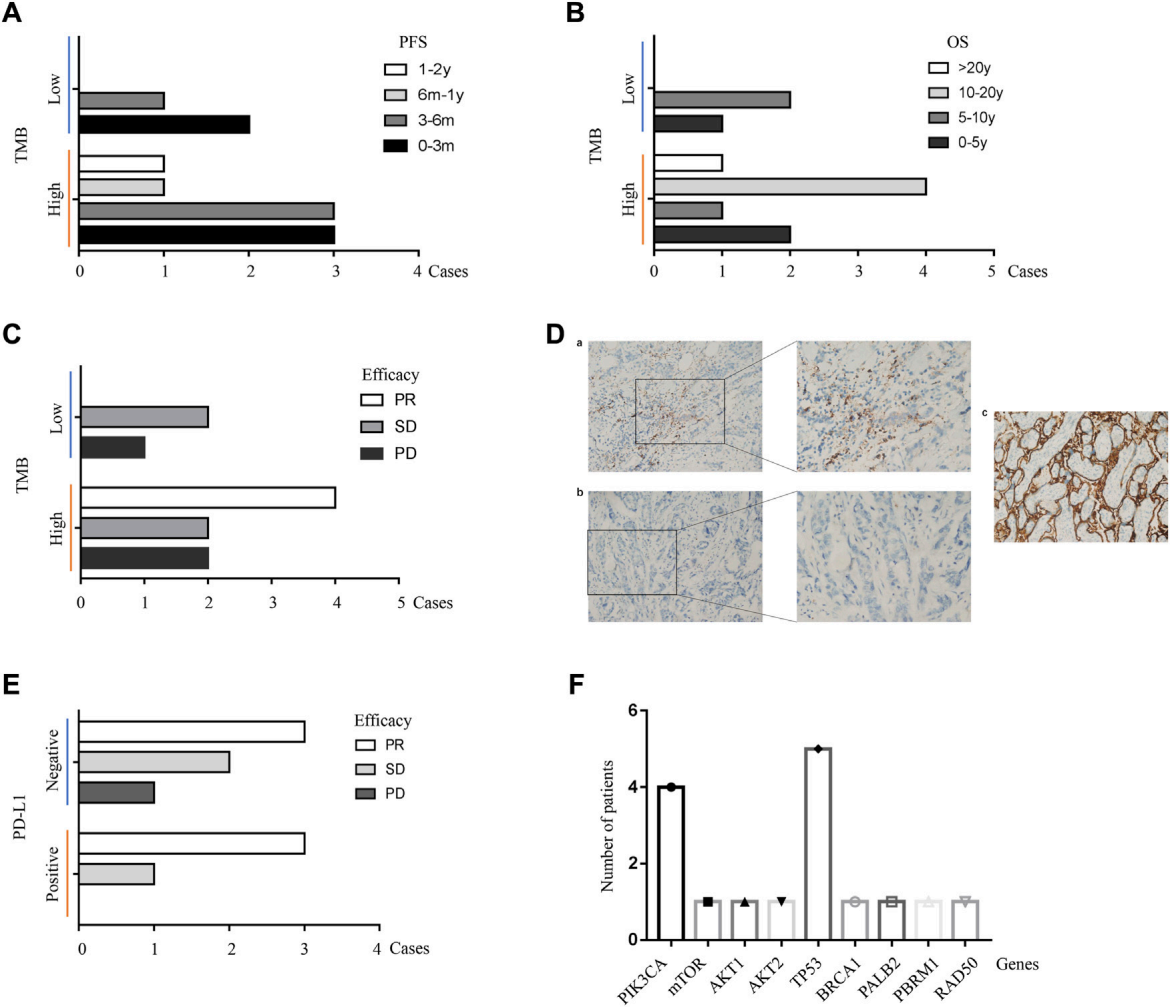
Alterations of genes in patients with mTNBC. **(A)** The progression-free survival was analyzed from TMB-low carrier and TMB-high carrier. Obvious benefit of PFS was detected in patients with TMB-high tumor; however, a significant difference was not reached (*p* > 0.05, Chi-squared analysis). The cases of patients were shown on the X-axis. Two levels of tumor mutational burden including TMB-low and TMB-high were demonstrated on the Y-axis. For each row, different periods of PFS were indicated with different colors. TMB, tumor mutational burden; TMB-high (TMB≥10.0 Muts/Mb); TMB-low (TMB<10.0 Muts/Mb); PFS, progression-free survival; 0–3 m, 0–3 months; 3–6 m, 3–6 months; 6 m-1 y, 6 months to 1 year; 1–2 y, 1–2 years. **(B)** The overall survival of patients treated with sintilimab was not affected by different statuses of tumor mutational burden. No significant difference of OS was found in the TMB-low and TMB-high groups (*p* > 0.05, Chi-square analysis). The cases of patients were shown on the X-axis. Two levels of tumor mutational burden including TMB-low and TMB-high were demonstrated on the Y-axis. For each row, different periods of OS were indicated with different colors. TMB, tumor mutational burden; TMB-high (TMB≥10.0 Muts/Mb); TMB-low (TMB<10.0 Muts/Mb); OS, overall survival; 0–5 y, 0–5 years; 5–10 y, 5–10 years; 10–20 y, 10–20 years; >20 y, more than 20 years. **(C)** The efficacy of sintilimab was not influenced by different statuses of tumor mutational burden. No significant difference of efficacy was detected in TMB-high carrier and TMB-low carrier (*p* > 0.05, Chi-squared analysis). The cases of patients were shown on the X-axis. Two levels of tumor mutational burden including TMB-low and TMB-high groups were demonstrated on the Y-axis. For each row, different efficacy of treatment with sintilimab including PR, SD, and PD was exhibited with different colors. TMB, tumor mutational burden; TMB-high (TMB≥10.0 Muts/Mb); TMB-low (TMB<10.0 Muts/Mb); PR, partial response; SD, stable disease; PD, progressive disease. **(D)** Immunohistochemistry results of PD-L1 staining. **(a)** Positive expression of PD-L1 (20×, 40×), **(b)** negative expression of PD-L1 (20×, 40×), and **(c)** positive control. **(E)** Patients with PD-L1-positive tumor gained a more favorable treatment response of sintilimab including PR and SD. However, significant difference was not reached (*p* > 0.05, Chi-square analysis). Unfavorable treatment response, PD, was only found in patients with PD-L1-negative tumor. The cases of patients were shown on the X-axis. Two expressed levels of PD-L1 including PD-L1-positive and PD-L1-negative were demonstrated on the Y-axis. For each row, different efficacies of treatment with sintilimab including PR, SD, and PD were shown with different colors. PD-L1, programmed death ligand-1; PR, partial response; SD, stable disease; PD, progressive disease. **(F)** Gene mutations were summarized from patients with advanced TNBC. The most frequent mutations were TP53 and PIK3CA. Other gene mutations included mTOR, AKT1, AKT2, BRCA1, PALB2, PBRM1, and RAD50. Mutational genes were exhibited on the X-axis. The number of patients was shown on the Y-axis.

Immune checkpoint inhibitors (ICIs) targeting PD-1 or its ligand PD-L1 had expanded the treatment landscape against breast cancer but were effective in only a subset of patients. Tumor mutational burden (TMB) was considered a promising tool to predict ICI-responsive tumor, especially for TNBC (O’Meara and Tolaney, 2021). It was reported that patients with TMB-high TNBC derived particular benefit from ICI in combination with chemotherapy (IMpassion130) or even ICI alone (KEYNOTE-119) (Schmid et al., 2020b; Winer et al., 2021). In this study, TMB was evaluable in 11 cases of patients (Table 4). In addition, 72.7% of patients (8/11) had TMB-high (TMB≥10.0 Muts/Mb) TNBC. Patients with TMB-high cancer were defined as the TMB-high carrier, while patients with TMB-low cancer, as the TMB-low carrier. Compared to TMB-low carriers, although TMB-high carriers treated with sintilimab did not reach the statistical significance of PFS and OS, however, the tendency of prolonged progression-free survival and overall survival was obvious, as shown in Figures 5A, B (*p* > 0.05). In addition, there was no significant difference in the efficacy of sintilimab between TMB-high carriers and TMB-low carriers (*p* > 0.05, Figure 5C), even though the trend is easily recognized. A specific association was indicated between the TMB status and improvement of treatment outcomes with sintilimab, but it may take a large amount of population to make the further verification.

**TABLE 4:** Molecular signature of patients with advanced TNBC.

Molecular signature	Patients evaluable, No. (%)
BRCA1/BRCA2 mutation	1/9 (11.1%)
Alterations of HRD genes	1/9 (11.1%)
MSS status	7/7 (100%)
TMB-high	8/11 (72.7%)
PD-L1 positive	4/10 (40%)

PD-L1-positive expression in tumor-infiltrating immune cells of 1% or more was considered to be associated with a better outcome treated by the PD-L1 inhibitor (Emens et al., 2019). In the phase III IMpassion130 study, clinical benefit from atezolizumab combined with nab-paclitaxel was reported to be driven by the PD-L1 IC + population (Schmid et al., 2018). In this study, tumor samples of 10 patients with advanced TNBC were available for detection of PD-L1 expression. In this small population, PD-L1-positive tumor was found in four cases (40%) of patients (Figure 5D). The correlation between PD-L1 expression and the efficacy of sintilimab was explored in this study. As shown in Figure 5E, patients with PD-L1-positive tumor gained a better treatment response; however, a significant difference was still not reached (*p* > 0.05). In conclusion, TMB-high and PD-L1-positive could be valuable markers for predicating therapeutic outcomes treated by sintilimab in TNBC.

Microsatellite instability (MSI) was caused by defective DNA mismatch repair (dMMR) genes and was characterized by a decrease or increase in repeated nucleotide sequences, which led to evasion of apoptosis, development of malignant mutations, and tumorigenesis (Luchini et al., 2019). Seven cases of patients had evaluable data about the status of MSI in this study; however, all their MSI status was found to be microsatellite-stable (MSS).

Genotyping was available in nine cases of patients with advanced TNBC. Homologous recombination repair (HRR) was a vital process for repairing DNA double-strand breaks. Germline variants of homologous recombination deficiency (HRD) genes, such as *BRCA1*, *BRCA2*, *ATM*, *BARD1*, *BRIP1*, *CHEK2*, *NBS1*, *PALB2*, *RAD51C*, and *RAD51D*, led to inherited susceptibility to specific types of cancer including breast cancer. It was reported that tumors with HRD had a distinct tumor microenvironment, including higher mutational burden and immunogenicity, suggesting that HRD may be a biomarker of the response to ICIs (Kang et al., 2023). As shown in Figure 5F, nine genes, namely, *TP53*, *PIK3CA*, *mTOR*, *AKT1*, *AKT2*, *BRCA1*, *PALB2*, *PBRM1*, and *RAD50* were the most frequent mutations detected in this study, which affected several cancer-related signaling pathways such as the PI3K/AKT/mTOR pathway and homologous recombination pathway. Among them, as shown in Table 4, 11.1% of patients (1/9) were found to have pathogenic BRCA1 germline mutation or PALB2 mutation, which was associated with HRD in breast cancer.

## Discussion

Breast cancer is the most diagnosed malignant neoplasm among Chinese females in 2020 (Tao et al., 2023). Compared to other subtypes of breast cancer, TNBC is a special subtype with clinical features including high metastatic potential, proneness to relapse, and poor prognosis. Brain and visceral organs are the common involved metastatic sites. Greater than 50% of patients experience a relapse in the first 3–5 years after diagnosis. Due to its special molecular phenotype, chemotherapy is the main systemic treatment, but the efficacy of conventional chemoradiotherapy is not well-satisfied. The residual metastatic lesions will eventually lead to tumor recurrence. Bevacizumab has been used in combination with chemotherapy to treat TNBC in some situation; however, the survival data did not increase significantly. The median survival after metastasis is 13.3 months, and the recurrence rate after surgery is as high as 25%. Therefore, it is urgent need to develop new treatment strategy for advanced TNBC.

In recent years, immune checkpoint inhibitors targeting PD-1 and PD-L1 have been widely used in the treatment of various solid tumors including breast cancer, especially TNBC, which is a breakthrough in the field of cancer therapy. TME is associated with suppression of the immune system and escaping of immune detection in TNBC. Co-inhibitory receptors including CTLA4 and PD1 have indispensable functions in modulation of immune responses (Duan et al., 2018). PD-1 binds to PD-L1 and transmits signals to inhibit T-cell proliferation and promote T-cell depletion. It was reported that 59% of TNBC patients highly expressed PD-L1, 70% of patients had high PD-1 expression, and 45% of patients had high expression of both PD-L1 and PD-1 (Khosravi-Shahi et al., 2018). Tumor cells can evade recognition and destruction by the host immune system through the immune checkpoint system; thus, blocking the immune checkpoint system is a promising treatment strategy for achieving effective antitumor immunity. TNBC is commonly assumed as suitable for immunotherapeutic treatments. In 2019, the US FDA granted accelerated approval of atezolizumab, a PD-L1 inhibitor, in combination with nab-paclitaxel for unresectable locally advanced or metastatic PD-L1-positive TNBC, based on the results of the phase III IMpassion130 trial, which represented the earliest immune checkpoint blockade (ICB) monoclonal antibody (mAb) approved for TNBC. In 2020, pembrolizumab, a PD-1 inhibitor, in combination with chemotherapy for locally recurrent unresectable or mTNBC patients with positive PD-L1 expression (CPS ≥10) was also approved by the FDA, based on results of the phase III KEYNOTE-355 trial (Heeke and Tan, 2021). However, the efficacy and safety of sintilimab have not been explored in advanced TNBC yet.

In this study, 87.5% of cases received neoadjuvant or adjuvant therapy and most of patients were heavily pretreated for several lines during the advanced stage of TNBC. Although 60% of patients received at least three lines of therapy and 20% received at least five before sintilimab administered, the median duration of response was still acceptable with 2.8 months (range, 0.7–21 months). The ORR and DCR of sintilimab-treated patients with mTNBC were 22.5% and 72.5%, respectively. The efficacy of sintilimab showed an “all-or-none” feature, which meant patients with sintilimab-sensitive tumor exhibited long-lasting PR or SD to the immunotherapy. However, for patients with resistant tumor, breast cancer progressed soon.

To uncover the impact from characteristics of the patient or tumor, correlation analysis was performed in this study. It was indicated that old age was significantly related to favorable treatment outcomes (*p* < 0.05). Outside the context of cancer, the elder patients were generally observed to have less effective immune responses to disease. Loss of T-cell receptor (TCR) diversity, decreased capacity of cytotoxic cells, and increased inflammatory signaling have been identified as age-related immune changes. Compared to younger patients with breast cancer, the elder patients were associated with fewer TNBC and HER2-positive subtypes and more luminal tumors. Favorable ICB biomarkers were found to be more prevalent in elder patients. Moreover, TMB was discovered to increase significantly with the patient age at diagnosis. All the above was demonstrated to promote the efficacy of immune checkpoint blockade in the elder population (Presley et al., 2021; Van Herck et al., 2021). The above information from the literature reports was consistent with our finding. The treatment efficacy of sintilimab was not affected by molecular markers from primary BC including HER2 expression, HR status, and the Ki67 level. All patients enrolled were pathologically confirmed as TNBC from metastatic tumors, regardless of different subtypes of primary tumors. Compared to other subtypes of breast cancer, TNBC is a special subtype with clinical features including high metastatic potential, proneness to relapse, poor prognosis, and immunotherapeutic benefit. In this study, we aimed to explore the influence from different subtypes of primary BC on the efficacy of sintilimab during the metastatic stage. Based on our small population, TNBC was shown to be more suitable for treatment of sintilimab compared with other subtypes of primary tumors; however, significant difference was not reached (*p* > 0.05).

In the IMpassion130 trial, patients with TNBC who had not received treatment in the metastatic setting were randomized to atezolizumab or placebo plus albumin-bound paclitaxel. Patients with PD-L1-expressed tumors significantly improved PFS (7.5 vs. 5 months; HR, 0.62; 95% CI, 0.49–0.78) and OS (25 vs. 15.5 months; HR, 0.62; 95% CI, 0.45–0.86) with therapy of ICI (Schmid et al., 2018). It was demonstrated that PD-L1-positive patients treated with conventional chemotherapy combined with immunotherapy as first-line therapy had a good prognosis (Schmid et al., 2020a). Based on the NCCN guideline, the primary goals of systemic treatment of advanced breast cancer are palliating symptoms, prolonging survival, and maintaining or improving quality of life. In this study, all patients enrolled in this study received the combination treatment of sintilimab and chemotherapy. Before the application of sintilimab, most patients have already used several commonly chemotherapeutic drugs including anti-angiogenesis, taxane, platinum, capecitabine, vinorelbin, gemcitabine, anthracyclines, cyclophosphamide, eribulin, and etoposide. The median PFS of sintilimab was 3.5 months (range, 1.4–21.0 months), with a 6-month PFS rate of 15.0% (6/40). Since 60% of cases received at least three lines of therapy for metastasis of breast cancer, the result of PFS observed in this study was understandable. The median OS was 52.5 months (range, 9.0–247.0 months), with 5-year and 10-year OS rates of 47.5% (19/40) and 22.5% (9/40), respectively. Based on the favorable efficacy and acceptable adverse effect, the prognosis (OS) was obviously prolonged in patients with advanced TNBC. It was regretful that because of the small PD-L1 evaluable population, the comparison of prognosis from different PD-L1-expressed tumors was not possible to perform in this study. A large amount of population may be induced into further study for investigating about the relationship between PD-L1 expression and treatment outcomes including efficacy and prognosis. In addition, the acceptable safety profile was monitored in the administration of sintilimab. The adverse effects of most patients were grade 1 to grade 2. Fatigue, skin rash, and pruritus were frequently observed in the study. Only one patient suffered from grade 3 treatment-related AEs including immune-mediated hepatitis and suppurative cholangitis, accompanying with an elevated liver function. However, all adverse effects recovered after the standardized treatment, especially dependent on the appropriate use of steroids. Our results provided an alternative therapy strategy and strengthened the confidence to apply sintilimab for patients with heavily pretreated mTNBC.

TNBC is a highly heterogeneous cancer with unique TME including special mutations and aberrant regulation of immunity. It is suitable for immunotherapy mainly due to tumor immune infiltration, neoantigens caused by mutational burden and higher genomic instability, and high levels of immune markers, which are closely correlated with tumor response, relapse, and overall outcomes. In this study, based on evaluated TMB data in 11 cases of patients, better prognosis of PFS and OS was found in TMB-high carrier. Similarly, to the literature of other ICIs reported, patients with PD-L1-positive tumor in this study gained more favorable treatment response from sintilimab than patients with PD-L1-negative tumor. It was suggested that TMB status and PD-L1 expression could be potential markers for predicting prognostic and effective benefit from treatment of sintilimab, but it may take a large amount of population to make the further verification. For the analysis of microsatellite instability, no patient was found with MSI-high metastatic breast cancer. Moreover, two patients were shown to have mutation of HRD genes (BRCA1 and PALB2). Even for patients with TNBC, the mutations of TP53 (12.5%, 5/40) and PIK3CA (10%, 4/40) gene were more frequently detected compared to other genes.

Considering the heterogeneity and malignancy of TNBC, multiple therapeutic approaches and combinations of regimens are essential to improve its treatment outcome. Until now, there is no recommendation of treatment for patients with advanced TNBC who have been previously treated for several lines in the metastatic setting. So immunotherapy is an inevitable trend for treatment of advanced TNBC. Based on our study, the immune checkpoint PD-1 inhibitor sintilimab offered satisfied benefits and acceptable adverse effects, which provided a novel therapeutic strategy for patients with heavily pretreated mTNBC.

## Data Availability

All original data presented in the study is included in the article; further inquiries can be directed to the corresponding author.
